# Sarcopenia in children with chronic liver disease: Prevalence and impact on liver transplant outcomes

**DOI:** 10.3389/fped.2022.1033570

**Published:** 2022-10-18

**Authors:** Silvio Veraldi, Andrea Pietrobattista, Giovanna Soglia, Lidia Monti, Tommaso Alterio, Antonella Mosca, Daniela Liccardo, Maria Sole Basso, Claudia Della Corte, Luca Russo, Manila Candusso, Fabrizio Chiusolo, Francesca Tortora, Marco Spada, Giuseppe Maggiore

**Affiliations:** ^1^ Hepatology, Gastroenterology, Nutrition, and Liver Transplantation Unit, Bambino Gesù Children's Hospital IRCCS, Rome, Italy; ^2^ Department of Anatomical, Histological, Forensic and Locomotor Apparatus Sciences, Sapienza University of Rome, Rome, Italy; ^3^ Department of Radiology, Bambino Gesù Children's Hospital IRCCS, Rome, Italy; ^4^ Department of Diagnostic Imaging, Oncological Radiotherapy and Hematology, A. Gemelli University Hospital Foundation IRCSS, Rome, Italy; ^5^ Anesthesia and Critical Care Medicine, Bambino Gesù Children's Hospital IRCCS, Rome, Italy; ^6^ Division of Hepatobiliopancreatic Surgery, Liver and Kidney Transplantation, Bambino Gesù Children's Hospital IRCCS, Rome, Italy

**Keywords:** chronic liver disease, liver transplantation, sarcopenia, frailty, pediatric patients

## Abstract

Sarcopenia is a clinical condition characterized by a reduction in muscle mass, which typically affects adult patients; however, it has recently been recognized in pediatric literature. Few studies in children with chronic liver disease (CLD) undergoing liver transplantation (LT) have investigated the role of sarcopenia, with controversial results. The aim of our study was to assess the prevalence and impact of sarcopenia among children with CLD who are candidates for LT. We conducted a retrospective, single-center study at Bambino Gesù Children's Hospital (Rome, Italy) from July 2016 to July 2021, evaluating all children (0–16 years old) with CLD listed for LT with an abdomen computed tomography imaging available before LT. The total psoas muscle surface area (t-PMSA) was defined as the sum of left and right psoas muscle surface area measured at L4–L5 on axial images. The t-PMSA *z*-score was calculated according to reference data, and sarcopenia was defined as a t-PMSA *z*-score of ≤−2 (1–16 years) or a psoas muscle index [PMI; PMI = t-PMSA/(100 × BSA)] of <50th percentile of the population examined (<1 year). Clinical, laboratory, and LT outcome data were collected from all the patients with CLD. 27 out 48 (56%) of the patients aged 1–16 years were sarcopenic. No differences were noted in anthropometrics, nutritional support, liver function tests, model for ESLD (MELD), or pediatric ESLD (PELD) scores between patients with and without sarcopenia. The former showed a higher prevalence of respiratory complications (66.7% vs. 42.1%) and need for inotropes (40.7% vs. 10.8%) after LT. Among patients aged 0–1 years (*n*: 36), those with reduced muscle mass (50%) had a longer hospitalization time (44 vs. 24 days) and higher incidences of multi-organ failure syndrome (38.9% vs. 0%) and intensive care unit-related infections (61.1% vs. 27.8%) compared to those with greater muscle mass. t-PMSA and PMI were statistically significant predictors of LT outcomes. Sarcopenia is a reliable index of frailty in children with CLD, as its presence is associated with the risk of a more challenging LT. Future studies will have to investigate the functional aspects of sarcopenia and conceive preventive measures of muscle wasting in CLD patients.

## Introduction

Chronic liver disease (CLD) in pediatric patients is associated with progressive structural and functional deterioration of the liver due to fibrosis, cholestasis, and subsequent hepatocellular necrosis (1). The nutritional state is a key element in pediatric patients with CLD, as it can affect the quality of life, morbidity, and mortality in both the pre- and post-transplant periods (2). Sarcopenia is a clinical condition characterized by the reduction of muscle mass and function and has been shown to play a relevant role in malnutrition (3, 4). Sarcopenia typically affects older or adult patients with chronic diseases, but it has recently been recognized in the pediatric literature with a growing interest, especially in CLD (5–7). Overall, knowledge on sarcopenia is limited by the lack of uniform definitions and similarities of principles with malnutrition, which could lead to the co-occurrence of both clinical conditions with overlapping risk in children with growth failure, neurodevelopmental delay, and postoperative outcome (8, 9). Recently, the North American Working Group on Sarcopenia in Liver Transplantation claimed that most children are too young to perform functional tests assessing frailty, which could lead to an underestimation of the impact of this condition on this vulnerable population (10). Thus, muscle mass assessment is a cornerstone of sarcopenia evaluation in this vulnerable population, and cross-sectional imaging such as computed tomography (CT) to evaluate the psoas muscle surface area is now considered a feasible method that provides objective data to assess the psoas or skeletal muscle surface area in children (6, 9, 11).

Moreover, in adults with cirrhosis, there is substantial evidence that sarcopenia is associated with increased morbidity and mortality before and after liver transplantation (LT); thus, some authors have advocated the inclusion of sarcopenia in the criteria of organ allocation for LT (12, 13). Poor studies have investigated the prevalence of sarcopenia in children with CLD undergoing LT and its correlation with transplant outcomes (6, 14–19).

The aim of our study was to assess the prevalence of sarcopenia among children with CLD who are candidates for LT.

The secondary aim was to evaluate the impact of sarcopenia on selected transplant outcomes and identify the distinctive features of sarcopenic subjects.

## Materials and methods

### Population and design of the study

This was a retrospective, single-center study conducted at Bambino Gesù Children's Hospital (Rome, Italy), evaluating all children (0–16 years old) with CLD listed for LT from July 2016 to July 2021, with an abdominal CT scan performed and available up to 3 months before LT.

Abdominal CT was performed in all subjects as part of the LT assessment protocol adopted at our center.

**Figure 1:**
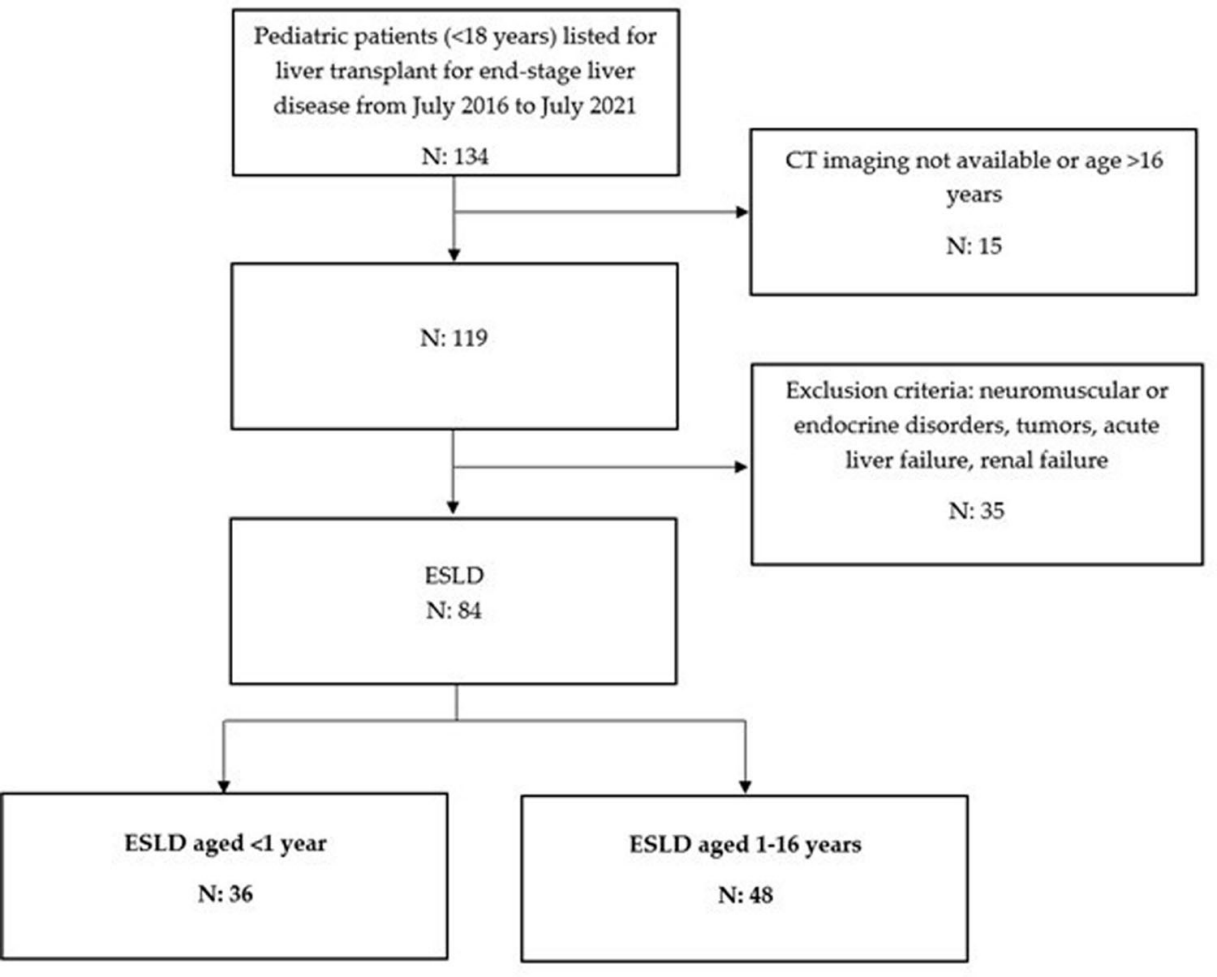
Enrolment flowchart of the population.

Patients candidates to LT without a chronic liver disease (acute liver failure) or with conditions that may impact on sarcopenia beyond the role of liver disease (i.e., chronic kidney disease, tumors, neuromuscular or endocrine disorders) were excluded (Figure 1).

Demographic (age, sex, main diagnosis, days on waiting list), anthropometric (weight, height, body surface area [BSA], pediatric end-stage liver disease (PELD)/model for end-stage liver disease (MELD) score), clinical (portal hypertension), and laboratory (full blood count, alanine transaminase, aspartate transaminase, gamma-glutamyl transferase, total bilirubin, international normalized ratio, serum albumin, vitamin D, alpha-fetoprotein, creatinine, and serum sodium) data were collected from patient charts. Laboratory results were registered only if they were performed within 1 month of CT imaging. Data on nutritional supplementation *via* a nasogastric tube (NGT), parenteral nutrition (PN), or both before LT were collected. The decision to start artificial nutrition was based on the clinical assessment of nutritional status (20). Early and late morbidities after LT represent the study outcomes.

The following outcomes were recorded during LT hospitalization: overall hospital and intensive care unit (ICU) length of stay (LOS); need for inotropes; need for re-intubation; Pediatric Index of Mortality 3 and Pediatric Organ Logistic Dysfunction 2 scores; and episodes of ICU infections and multiple organ dysfunction syndrome (MODS). MODS was defined as the presence of failure in at least two organs. ICU infections were defined as the presence of a positive bacterial culture in blood, urine, peritoneal fluid, or bronchoalveolar lavage detected during the ICU stay.

The incidence of sepsis, respiratory (acute respiratory distress syndrome, pneumothorax, pleural effusions, pneumonia), surgical (need for re-laparotomy, biliary or vascular complications), and graft-related (primary non-function, hepatic artery, or portal vein thrombosis) complications were recorded.

Finally, mortality, number of acute rejections based on the BANFF criteria (21), and catch-up growth in the first 12 months after LT were also considered.

Written informed consent to participate in this study was provided by the participants' legal guardian/next of kin. The study was carried out according to the rules of Helsinki declaration.

### Sarcopenia definition

**Figure 2:**
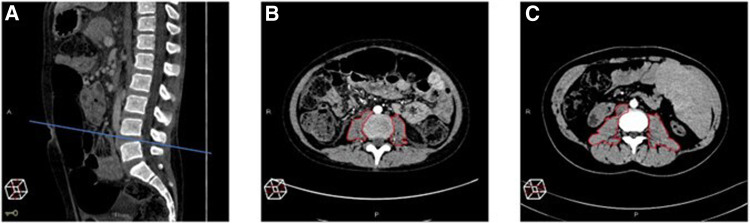
Measurement of the total psoas muscle surface area (t-PMSA). For the measurement of the total psoas muscle surface area (t-PMSA), the L4–L5 level in the sagittal plane is identified (**A**) then the left and right psoas muscle areas are measured in the axial plane (**B,C**). (**B**) Shows the psoas muscles of a subject with sarcopenia t-PMSA: 981.6 mm^2^, *z*-score: −2.88, while (**C**) those of a non-sarcopenic subject (t-PMSA: 2,390 mm^2^, *z*-score: 0.1).

CT scans were recovered using the Picture Archiving and Communication System and reviewed by an experienced radiologist (LM) with experience of over 20 years in abdominal radiology. The total psoas muscle surface area (t-PMSA) was defined as the sum of the left and right psoas muscle surface areas measured at L4-L5 on axial images. The t-PMSA *z*-score was calculated according to reference data, and sarcopenia was defined as a t-PMSA *z*-score of ≤−2 (Figure 2) (11).

To date, there is no standardized definition of sarcopenia for children aged <12 months as there are no reference values for t-PMSA and t-PMSA *z*-score available.

In this group, we calculated the psoas muscle index (PMI) as follows: PMI = (t-PMSA/(100 × BSA)) (mm^2^/(kg/m^2^)) and decided to stratify the population into two halves according to the median PMI. Infants with a PMI <50th percentile were conventionally considered to be sarcopenic.

### Statistical analysis

Continuous variables are expressed as median and 25th–75th centile or mean ± standard deviation, as appropriate. Categorical data are expressed as frequencies and percentages. Comparisons between continuous variables were performed by Mann–Whitney test or *t*-test. The chi-square test was used for proportions. Linear or binary logistic regression analyses were performed to study the t-PMSA *z*-scores or PMI (independent variables) and outcomes (dependent variables).

Statistical significance was set at *p* < 0.05. Statistical analysis was performed using SPSS v.22.0 (IBM Corp., Armonk, New York, United States).

## Results

Eighty-four children with CLD (41 males, 48.8%) who were listed for LT were evaluated. Thirty-six (16 males, 44%) were infants (<12 months old) and 48 (25 males, 52%) were aged 1–16 years old (Figure 1). Eighty-two of 84 (97.6%) patients underwent LT, while two were still on the waiting list at the end of the study. The most common indication for LT was biliary atresia (*n* = 60, 71%), followed by genetic disorders (*n* = 14, 16%) (Supplementary Table S1). Table 1 shows the demographic characteristics of the study population.

**Table 1:** Anthropometric characteristics of CLD population.

	CLD *n*: 84
Sex (M/F)	41/43
Age (months)	17.2 (6.5–71.5)
Weight *z*-score	−0.94 (±1.32)
Height *z*-score	−0.7 (±1.37)
PELD/MELD at WL	14 (5–20)
PELD/MELD at LT	14 (5–23)
Time on WL	70 (30–139)
PMI	9.18 (7.61–11.23)
t-PMSA (mm^2^)	431 (320–631)
Diagnosis (*n*, %)	BA: 60 (71.4%) Genetic: 14 (16.7%) Other: 10 (11.9%)

BA, biliary atresia; LT, liver transplantation; MELD, model for end-stage liver disease; PELD, pediatric end-stage liver disease; PMI, psoas muscle index; t-PMSA, total psoas muscle surface area; WL, waiting list.

Because of the lack of a validated definition of sarcopenia for children aged <12 months, we analyzed data from the CLD populations separately according to age.

### Population of 1–16 years

The prevalence of sarcopenia in this group was 56.3% (27 out 48). In patients with sarcopenia, the median t-PMSA *z*-score and mean PMI were −3.26 (±1.06) and 7.62 (6.12–8.67) compared to −0.737 (±0.989) and 10.73 (9.85–12.1), respectively (*p* < 0.001) (Table 2).

**Table 2:** Comparisons between sarcopenic and non-sarcopenic CLD patients.

	Population 1–16 years			Population 0–12 months		
	Sarcopenic *n*: 27 (56.3%)	Non-Sarcopenic *n*: 21 (43.7%)	*p*-value	PMI <50th centile *n*: 18 (50%)	PMI >50th centile *n*: 18 (50%)	*p*-value
Sex (M/F)	13/14	12/9	0.573	7/11	9/9	0.369
Age (months)	73.4 (21–119.1)	42.4 (19–120.9)	0.424	6 (5.3–8)	6.26 (5–9.1)	0.963
Weight *z*-score	−0.76 (±1.23)	−0.375 (±1.12)	0.128	−0.7 (±1.60)	−1.32 (±1.46)	0.285
Height *z*-score	−0.780 (±1.51)	−0.303 (±1.13)	0.255	−0.63 (±1.36)	−0.65 (±1.26)	0.636
Height *z*-score 1 year-after-LT	−0.363 (±1.80)	0.378 (±1.63)	0.255	−0.445 (±0.988)	−0.195 (±2.27)	0.382
PMI	7.62 (6.12–8.67)	10.73 (9.85–12.1)	<0.001*	7.82 (6.64–9.42)	11.25 (10.41–11.97)	<0.001*
t-PMSA (mm^2^)	518 (347–875)	699 (533–1208.5)	0.026*	272 (251–320)	387.5 (349.7–430.7)	<0.001*
t-PMSA *z*-score	−3.26 (±1.06)	−0.737 (±0.989)	<0.001*	N/A	N/A	–
PELD/MELD	8 (1–14)	5 (1–10)	0.124	20 (16–27)	20 (16–23)	0.743
Time on WL (days)	87.5 (18–194)	97 (74–356)	0.157	60 (31–86)	34 (18–51)	0.037*
Enteral nutrition (*n*, %)	3 (11.1%)	2 (10%)	1	7 (38.9%)	8 (44.4%)	1
Time on enteral nutrition (days)	255 (212–282)	212 (166–258)	0.789	45 (30–60)	18 (7–24)	0.128
Hemoglobin (g/dl)	10.0 (8.6–11)	10.9 (10.2–12.3)	0.011*	9.7 (8.7–10.3)	10.6 (9.3–11.2)	0.079
Platelets (nr/mm3)	114 (61–228.8)	129 (62–175)	0.914	182 (131–230)	228 (144–325)	0.097
AST (UI/L)	123 (56–249)	120 (67–174)	0.494	255 (149–345)	210 (164–270)	0.650
ALT (UI/L)	96 (37–132)	118 (60–155)	0.308	108 (58–198)	108 (58–198)	0.815
GGT (UI/L)	130 (61–250)	237 (80–467)	0.252	164 (59–334)	263 (148–627)	0.143
Total bilirubin (mg/dl)	8.7 (1.8–15.5)	3 (1.13–11.3)	0.119	20 (12.8–28.3)	14.8 (11.4–21.2)	0.355
INR	1.20 (1–1.4)	1.12 (1–1.40)	0.171	1.55 (1.39–1.82)	1.54 (1.43–1.77)	0.913
Albumin (g/dl)	3.1 (3–4)	3.8 (3.4–4.2)	0.039*	3 (2.8–3.1)	3.1 (2.8–3.6)	0.039
Na^+^ (mEq/L)	137 (135–138)	138 (136–140)	0.183	135 (134–137)	136 (135–137)	0.239
Creatinin (mg/dl)	0.25 (0.15–0.38)	0.22 (0.18–0.35)	0.855	0.16 (0.13–0.20)	0.16 (0.15–0.19)	0.839
Vitamin D (ng/ml)	26 (12–43)	31.5 (18.7–42)	0.366	26 (12–43)	31.5 (18.7–42)	0.786
Symptomatic portal hypertension (*n*, %)	18 (66.7%)	8 (38.1%)	0.031*	13 (72.2%)	12 (66.6%)	1
Diagnosis (*n*, %)	BA: 14 (51.8%) Genetic: 6 (22.2%) Other: 7 (26%)	BA: 11 (52.4%) Genetic 7 (33.3%) Other: 3 (14.3%)	0.599	BA: 17 (94.4%) Genetic: 1 (5.6%)	BA: 18 (100%) Genetic: 0 (0%)	1

BA, biliary atresia; LT, liver transplantation; PELD/MELD, Pediatric end-stage liver disease/Model for end-stage liver disease; PMI, psoas muscle index; t-PMSA, total psoas muscle surface area; WL, waiting list.

*p-value <0.05.

Patients with and without sarcopenia and CLD had similar weight and height *z*-scores at LT listing. In addition, the median growth increase one year after LT was comparable between the two groups.

There were no differences in the etiology of liver diseases, liver function tests, PELD/MELD score calculated at listing or LT, and median time spent on the waiting list. Patients with sarcopenia had a higher incidence of portal hypertension (*n* = 18, 66.7% vs. *n* = 8, 38.1%) and moderate-to-severe ascites than those without sarcopenia (*n* = 16, 59.3% vs. *n* = 5, 23.8%) (*p* < 0.05).

#### Impact of sarcopenia on transplant outcomes

Forty-six out of 48 (96%) patients with CLD aged 1–16 years old underwent LT during the study.

**Figure 3:**
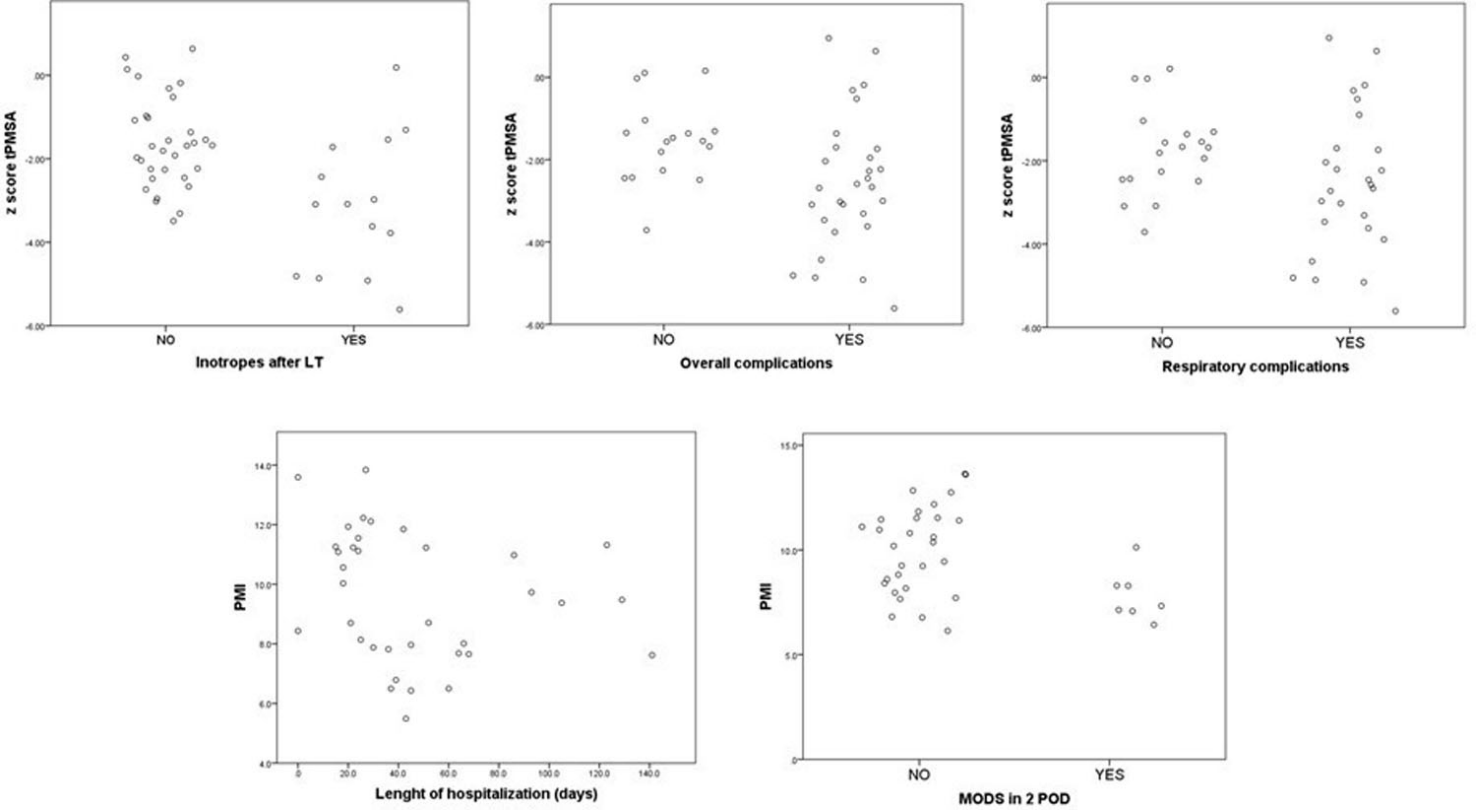
Correlations among *z*-score t-PMSA (1–16 years old) or PMI and outcomes after LT.

During ICU stay, the proportion of patients with inotropes after surgery was higher among those with sarcopenia (*n* = 11, 40.7% vs. *n* = 3, 10.8%) (Table 3) (Figure 3). The median duration of mechanical ventilation was similar between the two groups (12, 9.3–18.3 h vs. 13.1, 5.2–17.1 h), but children with sarcopenia more frequently failed to maintain noninvasive ventilatory support needing a second intubation (*n* = 8, 29.2% vs. *n* = 2, 10.5%).

**Table 3:** ICU outcomes in sarcopenic and non-sarcopenic patients.

	Population 1–16 years			Population 0–12 months		
	Sarcopenic *n*: 27	Non-sarcopenic *n*: 19	*p*-value	PMI <50th centile *n*: 18 (50%)	PMI >50th centile *n*: 18 (50%)	*p*-value
ICU stay (days)	4.0 (3–8)	5 (3.6–10.5)	0.483	18 (5–26)	7 (5–10)	0.026*
Inotropes during LT (*n*, %)	22 (81.5%)	13 (68.4%)	0.716	16 (88.9%)	15 (83.3%)	1
Inotropes after LT (*n*, %)	11 (40.7%)	3 (10.8%)	0.034*	10 (55.6%)	6 (33.3%)	0.315
ICU-related infections (*n*, %)	5 (18.5%)	4 (21%)	0.716	11 (61.1%)	5 (27.8%)	0.046*
Mechanical ventilation (hours)	13.1 (5.2–17.1)	12 (9.3–18.3)	0.980	21.3 (10.3–50.9)	17.5 (9.4–50.8)	0.719
Pa/FiO_2_ ratio before MV weaning	360 (275–406)	343 (282–381)	0.515	289 (200–411)	265 (213–339)	0.650
Need for NIV after MV weaning (*n*, %)	13 (48.1%)	8 (42.1%)	0.760	16 (89%)	14 (77.8%)	0.658
Need for re-intubation	8 (29.6%)	2 (10.5%)	0.090	4 (22.2%)	1 (5.6%)	0.338
CRRT (*n*, %)	1 (3.7%)	0 (0%)	1	4 (22.2%)	1 (5.6%)	0.338
PIM3	0.038 (0.024–0.078)	0.039 (0.031–0.208)	0.399	0.059 (0.043–0.204)	0.044 (0.031–0.065)	0.085
Pelod-2 at POD 1	5 (3.7–6.2)	5 (4–6.5)	0.768	5 (4–7)	4.5 (4–5.5)	0.252
Pelod-2 at POD 2	3 (1–5)	4 (2–5)	0.373	5 (4–7)	2.5 (2–4.2)	0.002*
No. of organ dysfunction at POD 1 (*n*, %)	7 (25.9%)	2 (10.5%)	0.230	8 (44.4%)	2 (11.1%)	0.060
No. of organ dysfunction at POD 2 (*n*, %)	3 (11.1%)	1 (5.2%)	0.496	7 (38.9%)	0 (0%)	0.008*
ICU mortality (*n*, %)	2 (7.4%)	0 (0%)	0.509	2 (11.1%)	1 (5.5%)	0.600

CRRT, continuous renal replacement therapy; ICU, Intensive care unit; LT, liver transplantation; MODS, multiple organ dysfunction syndrome; MV, mechanical ventilation; NIV, non-invasive ventilation: PELOD-2, pediatric logistic organ dysfunction 2; PIM3, pediatric index of mortality 3; POD, postoperative day.

*p-value <0.05.

No differences were observed in the incidence of MODS, ICU-related infections, or ICU mortality.

At the end of LT hospitalization, patients with sarcopenia showed an increased incidence of LT-related complications (*n* = 21, 77.8% vs. *n* = 8, 42.1%) (*p* < 0.05) (Table 4). In particular, a higher percentage of subjects had respiratory complications (*n* = 18, 66.7% vs. *n* = 8, 42.1%), while no differences were noted in surgical, infectious, and graft-related complications.

**Table 4:** Liver transplant-related complications in sarcopenic and non-sarcopenic patients.

	Population 1–16 years			Population 0–12 months		
	Sarcopenic *n*: 27	No-sarcopenic *n*: 19	*p*-value	PMI <50th centile *n*: 18 (50%)	PMI >50th centile *n*: 18 (50%)	*p*-value
Length of hospitalization (days)	25 (20–47)	23.5 (18.5–39)	0.553	44 (23–56)	24 (19–43)	0.039*
Overall complications (*n*, %)	21 (77.8%)	8 (42.1%)	0.028*	18 (100%)	16 (88.9%)	0.486
Respiratory complications (*n*, %)	18 (66.7%)	8 (42.1%)	0.063	18 (100%)	16 (88.9%)	0.486
Surgical complications (*n*, %)	6 (22.2%)	7 (36.8%)	0.332	7 (38.8%)	9 (50%)	0.724
Sepsis (*n*, %)	3 (11.1%)	2 (10.5%)	1	2 (11.1%)	0 (0%)	0.486
Graft failure HAT/PVT (*n*, %)	2 (7.4%)	3 (15.7%)	0.139	3 (16.7%)	1 (5.6%)	0.333
12-months acute rejections (*n*, %)	9 (33.3%)	9 (47.3%)	0.461	2 (11.1%)	6 (33.3%)	0.276
12-months survival (*n*, %)	23 (85.2%)	18 (94.7%)	0.369	13 (72.2%)	14 (77.8%)	0.704

HAT, hepatic artery thrombosis; PVT, portal vein thrombosis.

*p-value <0.05.

Patients with and without sarcopenia showed comparable ICU and overall LOS, as well as mortality and rate of acute rejections in the 12-months following LT.

Regression analyses revealed that the t-PMSA *z*-score was a significant predictor of the need for inotropes after surgery (odds ratio [OR] 0.487 [0.279–0.850], *p* < 0.05) and the risk of developing any complications [OR 0.535 (0.316–0.905), *p* < 0.05].

### Population of 0–12 months

We reviewed data from 36 infants (16 males; 6, 5.2–8.4 months) with CLD listed for LT with a median PMI of 9.5 (7.8–11.2) mm^2^.

To estimate the impact of sarcopenia in this population, we conventionally considered patients with sarcopenia and PMI of <50th centile (<9.5 mm^2^) and patients without sarcopenia those and PMI of ≥50th percentile (≥9.5 mm^2^).

As for older patients, infants with and without sarcopenia had similar anthropometric, clinical, and laboratory features (Table 2).

The proportion of children receiving enteral nutrition was comparable between the two groups (*n* = 7, 38.9% vs. 8, 44.4%), as was the duration of support.

Infants with sarcopenia tended to remain on the waiting list for a longer period (60, 31–86 vs. 34, 18–51 days), even if PELD scores at both listing and transplantation were similar between the two groups.

#### Impact of sarcopenia on transplant outcomes

Median ICU and overall hospitalization stays were longer in infants with sarcopenia (18, 5–26 and 44, and 23–56 days) than in those without (7, 5–10 and 24, and 19–43 days) (Tables 3, 4).

Moreover, patients with lower muscle mass presented a higher incidence of ICU-related infections (11 patients, 61.1% vs. 5 patients, 27.8%) and MODS both on the first (*n* = 8, 44.4% vs. *n* = 2, 11.1%) and second postoperative days (*n* = 7, 38.9% vs. *n* = 0, 0%) (Figure 3).

No differences in the rates of mortality and acute rejection in the first year after LT were found between the two groups.

Regression analysis revealed that PMI was a statistically significant predictor of length of hospitalization with a *β* coefficient of −4.303 [95% confidence interval (CI) −8.09 to −0.52] (*p* = 0.027) and MODS (OR: 0.513, 95% CI: 0.275–0.957) (*p* = 0.036) in the population of children aged 0–12 months.

## Discussion

Intervention on modifiable prognostic factors is part of the management of patient candidates for LT to improve their overall outcome. Recent literature has highlighted that sarcopenia is a common feature of adult patients with CLD, with a significant negative impact on both LT waitlist mortality and outcomes (22, 23).

Our study evaluated the prevalence and impact of sarcopenia in pediatric patients with CLD who were candidates for LT.

Various techniques are currently available for the assessment of skeletal muscle mass. Dual-energy x-ray absorptiometry and bioelectrical impedance analyses have been widely used in the past, but they may be affected by the patient's hydration status and lack reproducibility among different vendors or accuracy compared with other methods (24). Instead, abdominal CT scan and magnetic resonance evaluation of specific features of the psoas muscle (surface area measured at L3/L4/L5 or thickness) have proven to be reliable and reproducible with greater accuracy compared to other methods, especially in specific settings, such as patients with cirrhosis (24–27).

The main limitation of CT is the exposure to ionizing radiation, which is a major issue, mostly in pediatric patients. In our center, CT scan is performed in all patient candidates for LT as a part of the local listing protocol; therefore, we could retrospectively review the scans in our patients in order to assess skeletal muscle mass.

In our study population aged 1–16 years, we found a sarcopenia prevalence of 56%. This proportion is higher than those reported in previous studies, but comparable to that described in a recent Dutch study (55.6%) conducted on a population of 34 children with CLD (6, 14–19). Table 5 summarizes the results of the most recent reports on this topic.

**Table 5:** Pediatric studies on sarcopenia in children with end stage liver disease.

Reference	Radiological technique	Sarcopenia definition	Population	Prevalence of sarcopenia	Outcome
Jitwongwai et al. 2020 (14)	t-PMSA at L3/L4 in CT	PMI <50th of the population	105 LT (ESLD); <18 years	N/A	↑ LOS in hospital ↑ surgical complications (vascular)
Takeda et al. 2020 (15)	t-PMSA at L4/L5 in CT	t-PMSA <−2DS of the mean of a control population	89 infants with biliary atresia listed for LT	23.6%	↑ incidence of portal stenosis/thrombosis ↑ time of surgery (LT) ↑ duration of mechanical ventilation ↑ risk of sepsis
Verhagen et al. 2021 (16)	t-PMSA at L3/L4 in CT	t-PMSA *z*-score <−2DS sec. Lurz et al.	101 LT (ESLD); <18 years	55.6% (in the age group 1-16 years)	↑ LOS in hospital ↑ risk of infections
Boster et al. 2021 (17)	t-PMSA at L4/L5 in CT or RM	tPMA *z*-score <−2DS sec. Harbaugh et al.	57 LT (ESLD); <18 years	N/A	↑ mortality before and after LT
Woolfson et al. 2021 (18)	t-PMSA at L4/L5 in CT	t-PMSA *z*-score <−2DS sec. Lurz et al.	25 LT (ESLD); 1–16 years	40%	↑ need for enteral nutrition ↑ LOS in ICU ↓ statural growth
Dag et al. 2021 (19)	t-PMSA at L4/L5 in CT	N/A	276 LT (ESLD); <18 years	N/A	↑ LOS in hospital ↑ mortality
Lurz et al. 2018 (6)	t-PMSA at L3/L4 and L4/L5 in CT	N/A	25 ESLD listed for LT; <18 years	N/A	–

ESLD, end-stage liver disease; ICU, intensive care unit; LOS, length of stay; LT, liver transplantation.

Malnutrition is a known predictor of poor outcomes in children undergoing LT (19). However, currently available clinical and laboratory tools to accurately determine poor nutritional status in children are affected by organomegaly and ascites, among others. In our populations, we did not identify differences between subjects with and without sarcopenia regarding auxological parameters. Although muscle mass development and anthropometrics share some pathophysiological patterns, the lack of correlations between these variables may indicate that they are independently present in patients with CLD.

This finding is further supported by the same proportion of patients requiring artificial nutritional support regardless of the presence of sarcopenia. In 2021, Woolfson et al. highlighted that children with sarcopenia more frequently needed enteral or parenteral support (70%) than those without sarcopenia (15.6%); however, the population with sarcopenia also showed worse weight and height *z*-scores, which may explain the higher proportion of patients requiring nutritional support (18).

We suggest that t-PMA *z*-scores are a more sensitive and reliable marker for muscle mass and, therefore, are a more accurate method for assessing nutritional status in children with CLD.

The most recent guidelines for nutritional management of children with CLD are focused on advising an increased caloric intake to 130%–150% of dietary reference values but lack a clear indication on how to prevent or adjust nutritional support in case of sarcopenia (20). In this regard, the assessment of sarcopenia should be implemented during the evaluation of patients with CLD, which might become a novel indication for a dedicated nutritional regimen.

Our study highlighted that both patients with and without sarcopenia had similar PELD and MELD scores. In adults, the evidence that sarcopenia is associated with the onset of unfavorable outcomes, both before and after LT, led to the development of special MELD models corrected for including sarcopenia (“Sarco-MELD”) (12, 28, 29). The optimization of MELD with sarcopenia parameters remains controversial. Data from adult studies have shown that patients with sarcopenia are often burdened by prolonged and complicated hospitalizations, with increased mortality. This finding might ideally lead to the prioritization of allocation in this specific setting; however, the state of low functional reserve determined by sarcopenia may in itself impact pre- and post-transplant outcomes (30). Moreover, the presence of severe sarcopenia could burden the total score, and patients may be recommended LT at an early and compensated stage of liver disease. For these reasons, to date, Sarco-MELD is not widespread in routine clinical practice, and its transfer to the pediatric setting seems to have a narrow window.

By analyzing the outcomes after LT, we found that infants with lower muscle mass tended to be hospitalized for a longer period, especially in the ICU setting. This outcome was pointed out in previous studies; in particular, Jitwongwai et al. showed that the median hospital LOS after LT in patients with sarcopenia was longer than that in patients without (53 vs. 45 days) (14). Similarly, Verhagen et al. demonstrated a negative correlation between skeletal muscle index and overall (*r* = −0.3, *p* = 0.01) and ICU LOS (*r* = −0.3, *p* = 0.01) (16). In children, prolonged hospitalization is a typical feature of frailty and is associated with a significant increase in medical complications and economic costs, as has already been determined in adult studies (31, 32).

According to the European Working Group on Sarcopenia in Older People (EWGSOP), frailty is a multidimensional syndrome that increases vulnerability to poor health outcomes. It has been historically labeled as a “geriatric” condition as it is strictly related to aging, even if recent studies have begun to translate this concept in pediatric patients with chronic diseases (3, 33–35). Frailty encompasses both social and physical domains, with sarcopenia representing the main determinant.

In the analysis of LT outcomes, we found that patients with sarcopenia were more prone to developing complications (e.g., ICU infections, respiratory complications, and MODS) than patients without, while the presence of sarcopenia did not increase the risk of death or graft failure after LT. Similarly, previous pediatric studies demonstrated worse clinical evolution in sarcopenic children with various LT outcomes (e.g., risk of sepsis and vascular complications). Regarding mortality, only two works identified a correlation between t-PMSA and survival after LT (17, 19). In particular, Booster et al. described an increased risk of death by 60% per 100 mm^2^ decrease in PMSA in a population of 57 children (0–18 years old) listed for LT. Moreover, in our study, indices of sarcopenia (t-PMSA and PMI) were independent predictors of some LT outcomes; therefore, we may speculate that the presence of sarcopenia negatively impacts the evolution of LT, and thus is considered as a paradigm of frailty in CLD pediatric patients.

This study bears several limitations. First, it has a retrospective design. The timing of CT imaging was heterogeneous (<3 months before LT), and further variations in muscle mass may have occurred before LT. The secondary limitation is the lack of reference values for t-PMSA in children aged <12 months, as the only available data refer to a population aged 1–16 years old (11). Moreover, the current definition of sarcopenia also considers the functional impact of reduced muscle mass; however, to date, there are no standardized tests available to assess this aspect, especially in younger age groups (9).

In conclusion, reduced muscle mass is now recognized as a frequent feature of children and adolescents with CLD and is related to a more complicated post-transplant course. Early identification of sarcopenia is crucial to enable targeted treatment and prevention across pediatric clinical populations, especially for those requiring LT.

In the future, it will be necessary to identify new noninvasive, reliable, and reproducible techniques for diagnosing and monitoring muscle mass with widespread use in clinical practice.

## Data Availability

The raw data supporting the conclusions of this article will be made available by the authors, without undue reservation.
