# Supplementary material for: Sarcopenia in children with chronic liver disease: Prevalence and impact on liver transplant outcomes
Source: Front Pediatr. 2022 Oct 18;10:1033570. doi: 10.3389/fped.2022.1033570 (PMC9891232; doi:10.3389/fped.2022.1033570)
Supplement: Supplementary file 1 [file Table1.docx]

**Supplementary table 1 – Main diagnosis of the population (n, %)**

| **CLD**  n: 84 |
| --- |
| BA: 60 (71.4%)  Genetic: 14 (16.7%)  - Alagille syndrome: 6 (43%)  - Cystic fibrosis: 2 (14.3%)  - PFIC 2: 2 (14.3%)  - BAAT deficiency: 1 (7.1%)  - Delta 4-3-oxosteroid 5 beta-reductase deficiency: 1 (7.1%)  - Congenital hepatic fibrosis: 1 (7.1%)  - CD40 ligand deficiency: 1 (7.1%)  Other: 10 (11.9%)  - Cryptogenic cirrhosis: 4 (40%)  - Autoimmune sclerosing cholangitis: 2 (20%)  - Neonatal sclerosing cholangitis: 1 (10%)  - Budd-Chiari syndrome: 1 (10%)  - Chronic GVHD in BMT: 1 (10%)  - Hepatic adenomatosis: 1 (10%) |

BA: biliary atresia; BAAT: bile acid CoA:amino acid N-acyl transferase; BMT: Bone marrow transplantation; GVHD: Graft Versus Host Disease; LT: liver transplantation; MELD: model for end-stage liver disease; PELD: pediatric end-stage liver disease; PFIC: Progressive familial intrahepatic cholestasis PMI: psoas muscle index; tPMSA: total psoas muscle surface area; WL: waiting list;
